# Analysis of explicit model predictive control for path-following control

**DOI:** 10.1371/journal.pone.0194110

**Published:** 2018-03-13

**Authors:** Junho Lee, Hyuk-Jun Chang

**Affiliations:** 1 Department of Secured Smart Electric Vehicle, Kookmin University, Seoul, 02707, Republic of Korea; 2 School of Electrical Engineering and Department of Secured Smart Electric Vehicle, Kookmin University, Seoul, 02707, Republic of Korea; Chongqing University, CHINA

## Abstract

In this paper, explicit Model Predictive Control(MPC) is employed for automated lane-keeping systems. MPC has been regarded as the key to handle such constrained systems. However, the massive computational complexity of MPC, which employs online optimization, has been a major drawback that limits the range of its target application to relatively small and/or slow problems. Explicit MPC can reduce this computational burden using a multi-parametric quadratic programming technique(mp-QP). The control objective is to derive an optimal front steering wheel angle at each sampling time so that autonomous vehicles travel along desired paths, including straight, circular, and clothoid parts, at high entry speeds. In terms of the design of the proposed controller, a method of choosing weighting matrices in an optimization problem and the range of horizons for path-following control are described through simulations. For the verification of the proposed controller, simulation results obtained using other control methods such as MPC, Linear-Quadratic Regulator(LQR), and driver model are employed, and CarSim, which reflects the features of a vehicle more realistically than MATLAB/Simulink, is used for reliable demonstration.

## Introduction

In recent years, model predictive control(MPC) has become the standard optimization method for complex constrained systems because it can cope with such constraints and predict future events of a system. At each sampling time, an MPC controller solves an open-loop optimal control problem to obtain a sequence of optimal vectors, and this calculation is repeated at the next sampling time over a shifted horizon. Then, only the first input vector of the optimal input vectors is selected as the control action to the system, whereas the other optimal vectors are discarded.

Owing to its good performance in deriving an optimal control action fulfilling such complex constraints, MPC has been widely used in the automotive industry [1]. For example, in [2], MPC has been employed for vehicle yaw stability, where the constraint of yaw moments is caused by applying braking force to the wheels. Through simulations, it has been demonstrated that the proposed controller, which is designed based on an MPC scheme, can follow the desired reference values of yaw rate while fulfilling the constraints. Moreover, in [3], MPC has been applied to vehicle stabilization. In [3], state boundaries of the vehicle were defined to ensure that the vehicle motion remained within a stable region. The constraints indicate the physical limitations of the vehicle, e.g., the angular limit of handling and slip angle; moreover, the efficacy of the proposed controller in fulfilling the constraints has been demonstrated. MPC has been employed in [4] to stabilize an autonomous vehicle, and its capability has been demonstrated in a wind rejection scenario. In addition, linear-time varying MPC(LTV-MPC) and nonlinear MPC(NMPC) have been employed in autonomous vehicles in [5]. Furthermore, in [6], MPC has been applied for energy-saving vehicle to improve car-following fuel economy.

However, despite the aforementioned advantages of MPC, its huge computational complexity, which is caused by online optimization at each sampling time, is a huge drawback and limits its range of target applications to relatively small and/or slow systems. To overcome this limitation, a novel approach based on an MPC scheme that moves all the computational efforts offline has been proposed in [7], and this method is called *explicit* MPC.

In the explicit MPC scheme, a state vector is treated as a vector of parameters using a multi-parametric quadratic programming(mp-QP) technique. In this technique, a region contiaining a unique sequence of MPC feedback laws is presented as a piecewise affine function of the state, referred to as a critical region, and the controller *explicitly* selects one of these regions according to the state condition. It has been proved in [7] that explicit MPC can reduce the computational burden of MPC while preserving its performance.

In [8], explicit MPC has been applied to DC-DC switched-mode power supplies, and it has been demonstrated that explicit MPC shows adequate efficiency for use in industrial micro-controllers because it reduces the online computation power requirment. Moreover, this strategy has been employed to develope a robust MPC scheme in [9]. The mp-QP technique has been applied successfully for implementing MPC controllers, and it has been shown that the explicitly obtained control law ensures robust handling. This technique has also been used in active front steering(AFS) systems. In [10], because all optimization solutions were calculated offline using the mp-QP technique, the proposed MPC controller could execute at a high rate in an electronic control unit(ECU).

In this paper, explicit MPC is applied to the path-following control to be analyzed. In the past few decades, interest in path-following control for autonomous vehicles has increased significantly [11–16]. In the path-following maneuver, a vehicle is supposed to follow a desired path by minimizing deviations from the path. The controller steers the vehicle’s orientation to drive it along the path with an assumed constant longitudinal vehicle speed. In [11], a nested proportional integral differential(PID) steering controller has been designed for vision-based autonomous vehicles, where the look-ahead point for calculating the desired motion of a vehicle was determined by a vision system. PID control has been applied to obtain an optimal yaw rate, which served as the control input, and PI control has been used for controlling the AFS system. Moreover, for path-following in autonomous vehicles, a new MPC structure considering both kinematic and dynamic control has been proposed in [12]. The structure was of the cascade type, and at the kinematic level, the controller has been defined to reduce computational complexity and provide set points for the controller at the dynamic level. Furthermore, path-following control has been applied to tractor trailers [13]. In [13], a linear parameter-varying(LPV) controller has been designed to be dependent on longitudinal velocity, which varies according to the driving condition. In [14], the desired path was a type of double lane change and MPC-based approaches has been employed for predictive active steering control. A nonlinear vehicle model and the Pacejka tire model has been used to design a nonlinear MPC(NMPC) controller and a linear time-varying MPC(LTV-MPC) controller. Reduction in the computational complexity of NMPC and the robustness of LTV-MPC has been addressed in [14]. Path-following control has also been applied for a fully actuated marine surface vessel [15]. In [15], an integral terminal sliding mode based composite nonlinear feedback technique has been employed for a path following maneuver and demonstrated the tracking performance and the robustness of the proposed controller.

**Fig 1:**
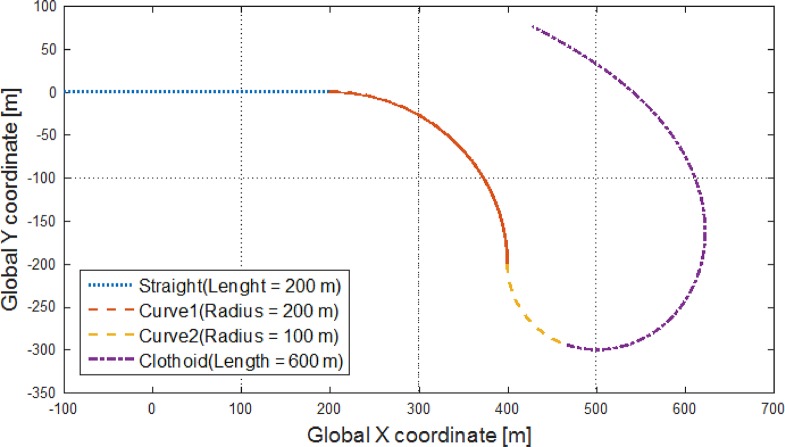
Desired path. Desired path for path-following control is plotted in this figure. This path comprises of four parts: a straight part, two curves, and a clothoid part. The straight part is used to prove the fulfilment of the proposed controller in this paper by setting the starting point, which is deviated from the desired path. By using the two curves and the clothoid part, the ability of the controller to perform path-following control will be demonstrated.

Moreover, in recent years, interest in path-following control for underactuated autonomous vehicles, which comprise systems with control inputs fewer than the number of degrees of freedom, has increased significantly. For example, in [16], a hybrid controller combining adaptive switching supervisory control with a nonlinear Lyapunov-based tracking control law has been proposed for path-following control of hover crafts and underwater vehicles. The concept of explicit MPC has been applied to yaw stabilization, leading to a demonstrable reduction in computational burden; this means that the proposed controller can be implemented in real time [17]. The main contribution of this paper is an analysis of explicit MPC from the veiwpoint of for path-following control so that this MPC-based control method can be more widely employed for not only automotive systems but also other smaller and/or faster systems. Moreover, by demonstrating the reduction of the computational complexcity of MPC, an explicit MPC controller can be designed on different types of devices from micro controller units(MCUs). Fig 1 shows a desired path comprising of a straight part, a curve, and a clothoid part. The curvature of the curve is constant, whereas the curvature of the clothoid part decreases linearly [18]. The vehicle model parameters refer to a vehicle model in CarSim(C-Class Hatchback 2017). For the analysis of the proposed controller, the weighting matrices, the prediction horizon, and the control horizon in the MPC optimization problem are varied, and the constant longitudinal velocity of the vehicle is changed. In addition, other optimization controllers such as the linear-quadratic regulator(LQR) and a controller applying the basic MPC concept are designed for comparing their performances with that of the proposed controller. Through simulations using MATALB/Simulink and CarSim, we demonstrate the ability of the proposed controller to fulfill such constraints while tracking the desired path.

## Vehicle model

In this section, the dynamics and the state-space representation of a vehicle model and the definition of vehicle parameters are presented.

**Fig 2:**
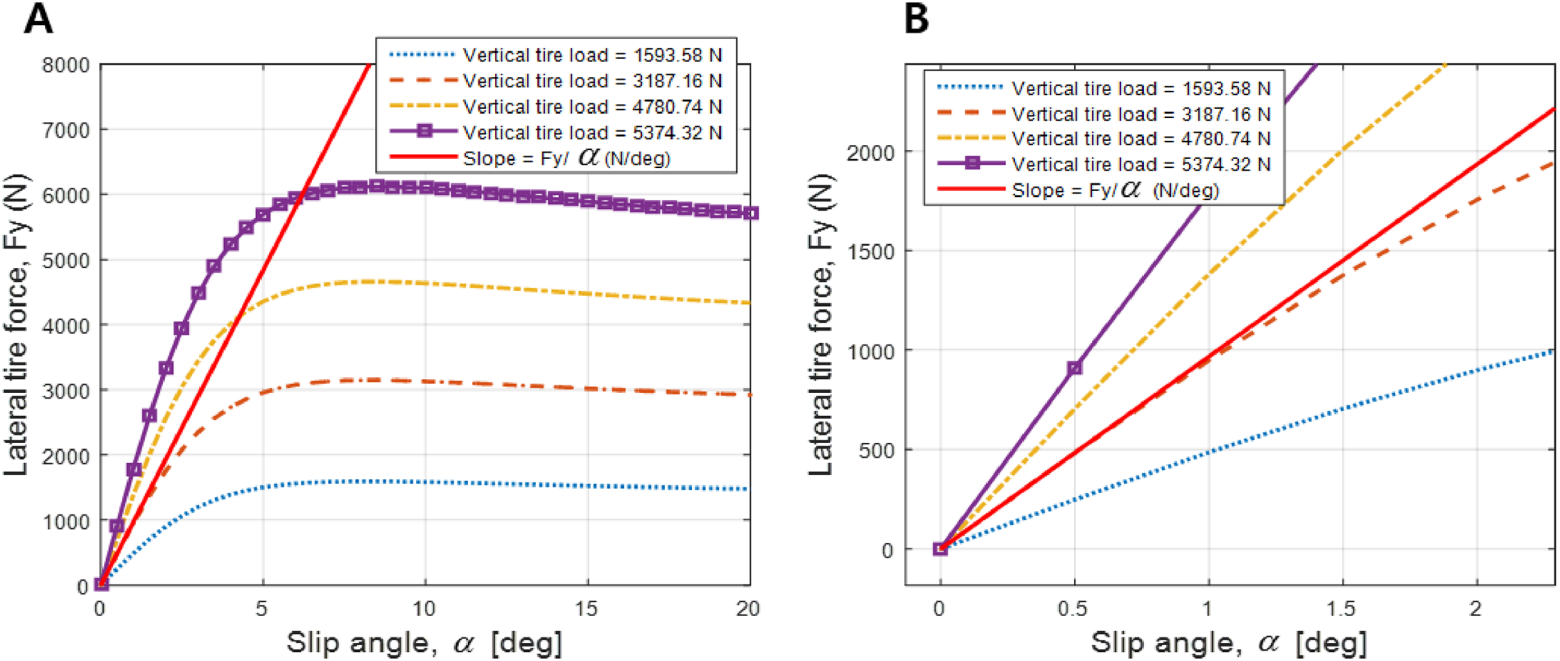
Tire cornering stiffness. (A) Corresponding lateral tire force, *F*_*y*_, as a function of the slip angle of the tire, *α* with different vertical tire loads. (B) the initial slope of the function (red line) when the vertical tire load is 3187.16 N, considering the vehicle mass, i.e., 967 N/deg or 55405 N/rad, which are both values of tire cornering stiffness *C*_*r*_ and *C*_*f*_, respectively.

Table 1 lists and quantifies the parameters of the path-following model for a vehicle model in CarSim(C-Class Hatchback 2017). Among these parameters, the front and the rear tire cornering stiffness values, *C*_*f*_ and *C*_*r*_, respectively, are not defined explicitly in CarSim because they vary according to tire slip angle. To use these values in a linear time-invariant system, the relationship between the lateral tire force *F*_*y*_ and the tire slip angle *α*, where *F*_*y*_ is a function of *α*, is used. Fig 2A shows a graph that can be used to calculate cornering tire stiffness, which is the initial slope of the graph. The tire model is 215/55 R17, and the slip ratio is 0.85. When the tire load is 3187.16 N, considering a vehicle mass of 1270 kg, the initial slope of the graph, plotted as a red line, is 967 N/deg or 55405 N/rad, as shown in Fig 2B. In this paper, it is assumed that the front and the rear tire stiffness values are the same.

**Table 1:** Parameters of vehicle model for path-following control.

Symbol	Description	Value[units]
*V*_*x*_	vehicle speed	20 [m/s]
*m*	vehicle mass	1270 [kg]
*a*	distance from center to front axis	1.015 [m]
*b*	distance from center to rear axis	1.895 [m]
*I*_*z*_	vehicle yaw inertia	1536.7 [kg ⋅ m^2^]
*C*_*f*_	front tire cornering stiffness	
*C*_*r*_	rear tire cornering stiffness	

This table identifies and quantifies the path-following control model parameters. The vehicle is assumed to move at a constant speed of 20 m/s, and other parameter values refer to a vehicle model in CarSim(C-Class Hatchback 2017). However, the values of front and rear tire cornering stiffness are not defined explicitly in CarSim. Therefore, these values are calculated in this paper using a function of the tire slip angle and the lateral force on the tire.

For path-following control, it is useful to set position and orientation errors as state variables. Accordingly, a dynamic model for path-following control can be expressed as follows [19]:

$$\begin{array}{ccc} m{\ddot{e}}_{1}\left(t\right) & = & {\dot{e}}_{1}\left(t\right)\left(-\frac{2C_{f}+2C_{r}}{V_{x}}\right)+e_{2}\left(t\right)\left(2C_{f}+2C_{r}\right)-{\dot{e}}_{2}\left(t\right)\left(\frac{2aC_{f}-2bC_{r}}{V_{x}}\right)\\ & & -{\dot{\psi }}_{des}\left(t\right)\left(\frac{2aC_{f}-2bC_{r}}{V_{x}}\right)+2C_{f}\delta \left(t\right)+mg\text{sin}\left(\phi \left(t\right)\right)\\ \text{and} & & \\ I_{zz}{\ddot{e}}_{2}\left(t\right) & = & 2aC_{f}\delta \left(t\right)-{\dot{e}}_{1}\left(t\right)\left(\frac{2aC_{f}-2bC_{r}}{V_{x}}\right)+e_{2}\left(t\right)\left(2aC_{f}-2bC_{r}\right)\\ & & -{\dot{e}}_{2}\left(t\right)\frac{2a^{2}C_{f}+2b^{2}C_{r}}{V_{x}}-I_{zz}{\ddot{\psi }}_{des}\left(t\right)-{\dot{\psi }}_{des}\left(t\right)\left(\frac{2a^{2}C_{f}+2b^{2}C_{r}}{V_{x}}\right), \end{array}$$ (1)

where *e*_1_(*t*), *e*_2_(*t*), *δ*(*t*), and sin(*ϕ*(*t*)) represent the lateral deviation of the mass center of the vehicle from the desired path, yaw angle deviation with respect to *ψ*_*des*_(*t*), desired yaw angle obtained from the desired path; front steering wheel angle, and road bank angle, respectively. The desired yaw rate is ${\dot{\psi }}_{des}\left(t\right)=\frac{V_{x}}{R(t)}$, where R is a radius of the desired path, and *g* is the gravitational acceleration.

A state-space representation of Eq (1), neglecting the influence of road bank angle, can be expressed as follows:

$$\begin{array}{ccc} \dot{x}\left(t\right) & = & Ax\left(t\right)+B_{1}u_{1}\left(t\right)+B_{2}u_{2}\left(t\right),\\ y(t) & = & Cx(t),\\ \text{where} & & \\ x(t) & = & {\left[\begin{array}{cccc} e_{1}\left(t\right) & {\dot{e}}_{1}\left(t\right) & e_{2}\left(t\right) & {\dot{e}}_{2}\left(t\right) \end{array}\right]}^{\prime },\;u_{1}\left(t\right)=\delta \left(t\right),\;u_{2}\left(t\right)={\dot{\psi }}_{des}\left(t\right),\\ A & = & \left[\begin{array}{cccc} 0 & 1 & 0 & 0\\ 0 & -\frac{2C_{f}+2C_{r}}{mV_{x}} & \frac{2C_{f}+2C_{r}}{m} & -\frac{2aC_{f}-2bC_{r}}{mV_{x}}\\ 0 & 0 & 0 & 1\\ 0 & -\frac{2aC_{f}-2bC_{r}}{I_{zz}V_{x}} & \frac{2aC_{f}-2bC_{r}}{I_{zz}} & -\frac{2a^{2}C_{f}+2b^{2}C_{r}}{I_{zz}V_{x}} \end{array}\right],\;B_{1}=\left[\begin{array}{c} 0\\ \frac{2C_{f}}{m}\\ 0\\ \frac{2aC_{f}}{I_{zz}} \end{array}\right],\\ B_{2} & = & \left[\begin{array}{c} 0\\ -\frac{2aC_{f}-2bC_{r}}{mV_{x}}-V_{x}\\ 0\\ -\frac{2a^{2}C_{f}+2b^{2}C_{r}}{I_{zz}V_{x}} \end{array}\right],\;C=\left[\begin{array}{cccc} 1 & 0 & 0 & 0 \end{array}\right]. \end{array}$$ (2)

The second input *u*_2_(*t*) is defined by the desired path as described in Fig 1. The control objective is to converge the output, *y*(*t*), to zero by the steering wheel angle, *δ*(*t*); i.e., the lateral position error of the vehicle with respect to the desired path converges to zero. In this paper, a zero-order hold method is applied with a sampling time of 0.01 s to employ Eq (2) in an explicit MPC scheme.

## Explicit model predictive control

In this section, the formulation of a basic MPC scheme and an introduction of explicit MPC are given.

### Formulation of model predictive control

This subsection explains a concept and a formulation of MPC.

MPC [20] has been employed as a key optimal control scheme to guarantee the robustness and fulfilment of such complex constraints [21]. Basically, at each sampling time, MPC solves an open-loop optimization problem with respect to the constraints [22]. The open-loop optimization problem can be defined as follows:

$$\begin{array}{ccc} & & \underset{U≜u_{t},...,u_{t+N_{u}-1}}{min}\{J(U,x(t)=\\ & & \sum_{k=0}^{N_{y}-1}[x_{t+k|t}^{\prime }Qx_{t+k|t}+u_{t+k}^{\prime }Ru_{t+k}\\ & & +{\left(y_{t+k}-y_{t+k}^{ref}\right)}^{\prime }QR\left(y_{t+k}-y_{t+k}^{ref}\right)]+x_{t+N_{y}|t}^{\prime }Px_{t+N_{y}|t}\},\\ \text{s.t.} & & x_{t|t}=x\left(t\right),\\ & & x_{t+k+1|t}=A_{d}x_{t+k|t}+B_{d}u_{t+k},\;k⩾0,\\ & & y_{t+k|t}=C_{d}x_{t+k|t},\\ & & u_{min}⩽u_{t+k|t}⩽u_{max},\;k=0,1,...,N_{u},\\ & & x_{min}⩽x_{t+k|t}⩽x_{max},\;k=0,1,...,N_{y},\\ & & u_{t+k}=Kx_{t+k|t},\;N_{u}⩽k<N_{y}, \end{array}$$ (3)

where *A*_*d*_, *B*_*d*_, and *C*_*d*_ are the discrete-time versions of the system, input, and output matrices in Eq (2), respectively, and *k* is the time index, i.e., $x\left(t\right)\in R^{n}$, and $u\left(t\right)\in R^{m}$. The notation *x*_*k*|*t*_ represents the value of *x*, which is predicted to be *k* steps ahead of *t*. *Q*, *R*, *P*, and *QR* are the weighting matrices for the state, input, and terminal state, respectively, at *k* = *N*_*y*_; moreover, the output with the corresponding dimensions and *P* can be obtained as the solution of the discrete-time algebraic Riccati equation as follows:

$$\begin{array}{ccc} P & = & A_{d}^{\prime }PA_{d}-A_{d}^{\prime }PB_{d}K+Q,\\ K & = & {\left(B_{d}^{\prime }PB_{d}+R\right)}^{-1}B_{d}^{\prime }PA_{d}, \end{array}$$ (4)

where *K* is the state-feedback gain matrix. It is assumed that P≽0, $Q=Q^{\prime }≽0$, and $R=R^{\prime }≽0$; moreover, *N*_*y*_ and *N*_*u*_ are the prediction horizon and the input horizon, respectively. For the MPC controller, *N*_*y*_ must be longer than or equal to *N*_*u*_ [23]. The constraints in Eq (3) are imposed on the state and the input along *N*_*y*_ and *N*_*u*_, respectively.

By solving Eq (3), a sequence of optimal input vectors *U* is obtained, and in the period of *N*_*u*_ ≤ *k* < *N*_*y*_, the MPC controller selects *K* as the optimal feedback gain matrix. Among the calculated input vectors, only the first input *u*_*t*_ is selected as the control action to the system, and the other input vectors are discarded. Then, the same task is repeated over a shifted horizon. Because *u*_*t*_ is the optimal control action at *t* = 0, which minimizes a cost function with respect to the prediction of the system along *N*_*y*_, MPC can predict future events of the system and fulfil constraints such as the ones mentioned in Eq (3).

Despite the advantages of MPC, repeated optimization at each sampling instance leads to considerable computational complexity; consequently, the range of possible target applications of MPC has been limited to relatively small and/or slow problems. To overcome this drawback, a new MPC-based approach has been suggested in [7]; this approach can solve the optimization problem *offline* by reformulating the problem as a multi-parametric quadratic program(mp-QP). A brief explanation of this approach is provided in the next section.

### Summary of explicit model predictive control

This subsection provides a summary of explicit MPC, and how optimization can be implemented offline. The main concept of explicit MPC is solving Eq (3) offline while sustaining the performance of MPC to expand its range of target applications to relatively larger or faster problems. Basically, an explicit MPC controller generates so-called *critical regions*, as piecewise affine functions of *x*(*t*), where a unique sequence of MPC feedback laws are defined in each critical region. Thereby, the controller explicitly selects a critical region that minimizes the cost function of mp-QP, which is transformed from the online optimization problem in Eq (3).

The prediction equations of the state vector $x\left(t\right)\left(\in R^{nN_{y}}\right)$ can be derived as follows:

$$\begin{array}{ccc} & & x(t)=\Theta x(t)+\Lambda u(t),\\ \text{where} & & \\ & & x\left(t\right)=\left[\begin{array}{c} x(k+1|k)\\ \vdots \\ x(k+N_{y}|k) \end{array}\right],u\left(t\right)=\left[\begin{array}{c} u(k|k)\\ \vdots \\ u(k+N_{y}-1|k) \end{array}\right],\\ & & \Theta =\left[\begin{array}{c} A_{d}\\ \vdots \\ A_{d}^{N_{y}} \end{array}\right],\;\Lambda =\left[\begin{array}{cccc} B_{d} & 0 & \cdots & 0\\ A_{d}B_{d} & B_{d} & \cdots & 0\\ \vdots & \vdots & \ddots & \vdots \\ A_{d}^{N_{y}-1}B_{d} & A_{d}^{N_{y}-2}B_{d} & \cdots & B_{d} \end{array}\right], \end{array}$$ (5)

where $u\left(t\right)\in R^{mN_{u}}$. Using Eqs (5) and (3) can be reformulated as follows:

$$\begin{array}{ccc} & & V\left(x\left(t\right)\right)=\frac{1}{2}x^{\prime }\left(t\right)Gx\left(t\right)+\underset{U}{min}\left\{\frac{1}{2}u{\left(t\right)}^{\prime }Hu\left(t\right)+x^{\prime }\left(t\right)Fu\left(t\right)\right\},\\ & & \text{s.t.}\;A_{c}u\left(t\right)⩽b_{0}+B_{c}x\left(t\right),\\ \text{where} & & \\ & & H=\Lambda^{\prime }\tilde{Q}\Lambda +\tilde{R},\;F=\Lambda^{\prime }\tilde{Q}\Theta ,\;G=\Theta^{\prime }\tilde{Q}\Theta +Q,\\ & & \tilde{Q}=\left[\begin{array}{cccc} Q & & & \\ & \ddots & & \\ & & Q & \\ & & & P \end{array}\right],\;\tilde{R}=\left[\begin{array}{ccc} R & & \\ & \ddots & \\ & & R \end{array}\right],\\ & & A_{c}=\left[\begin{array}{c} \Lambda_{i}\\ -\Lambda_{i} \end{array}\right],\;i=1,...,N_{y},\;b_{0}=\left[\begin{array}{c} x_{max}\\ -x_{min} \end{array}\right],\;B_{c}=\left[\begin{array}{c} -A_{d}^{i}\\ A_{d}^{i} \end{array}\right],\;i=1,...,N_{y}. \end{array}$$ (6)

In Eq (6), Λ_*i*_ indicates the *i*th row of Λ, $\tilde{Q}\in R^{nN_{y}\times nN_{y}},\;\tilde{R}\in R^{mN_{y}\times mN_{y}}$, and *H*, *F*, and *G* can be calculated offline.

By defining $z≜u\left(t\right)+H^{-1}F^{\prime }$, the re-written optimization problem in Eq (6) can be expressed as a mp-QP problem as follows [7]:

$$\begin{array}{ccc} & & V_{z}\left(x\right)=\underset{z}{\text{min}}\frac{1}{2}z^{\prime }Hz\\ & & \text{s.t.}\;A_{c}z⩽b_{0}+B_{z}x\left(t\right), \end{array}$$ (7)

where $V_{z}\left(x\right)=V\left(x\left(t\right)\right)-\frac{1}{2}x^{\prime }\left(G-FH^{-1}F^{\prime }\right)x$ and $B_{z}≜B_{c}+GH^{-1}F^{\prime }$. In Eq (7), *z*, which is obtained using the Karuch-Kuhn-Tucker optimality conditions [24], is a piecewise affine function of the state vector *x*(*t*). The inequality constraint in Eq (7) is a polytope; therefore, the generated critical regions are polytopes as well.

In this paper, we use the POP toolbox developed by Texas A&M University to design the proposed explicit MPC controller. A parametrized vector, denoted as *θ*, can be defined with respect to both/either the state vector, and the input vector or output vector (the reader can refer to [25] for more details). Using the POP solver, the inequality constraint in Eq (7) can be defined as follows:

$$\begin{array}{c} \theta \in R^{q}|CR_{A}\theta \leq CR_{b}, \end{array}$$ (8)

where *q* is the number of parameters. The critical regions are segmented based on Eq (8) by generating multiple of constraints *h* for each critical region. *CR*_01_ is the first and the largest critical region, and the remainder of the critical regions *CR*_*rest*_ can be defined using the POP solver as follows:

$$\begin{array}{ccc} & & CR_{j+1}=\{CR_{A}^{l}\leq CR_{b}^{l}\},\;l=1,...,h,\;j=1,...,N_{CR}-1,\\ \text{where}\;\;\;\;\;\;\;\;\;\;\;\;\;\;\; & & \\ & & h=1-4(n+p+q). \end{array}$$ (9)

In Eq (9), *n*, *N*_*CR*_, and *p* are the number of continuous variables, critical regions, and binary variables, respectively.

**Fig 3:**
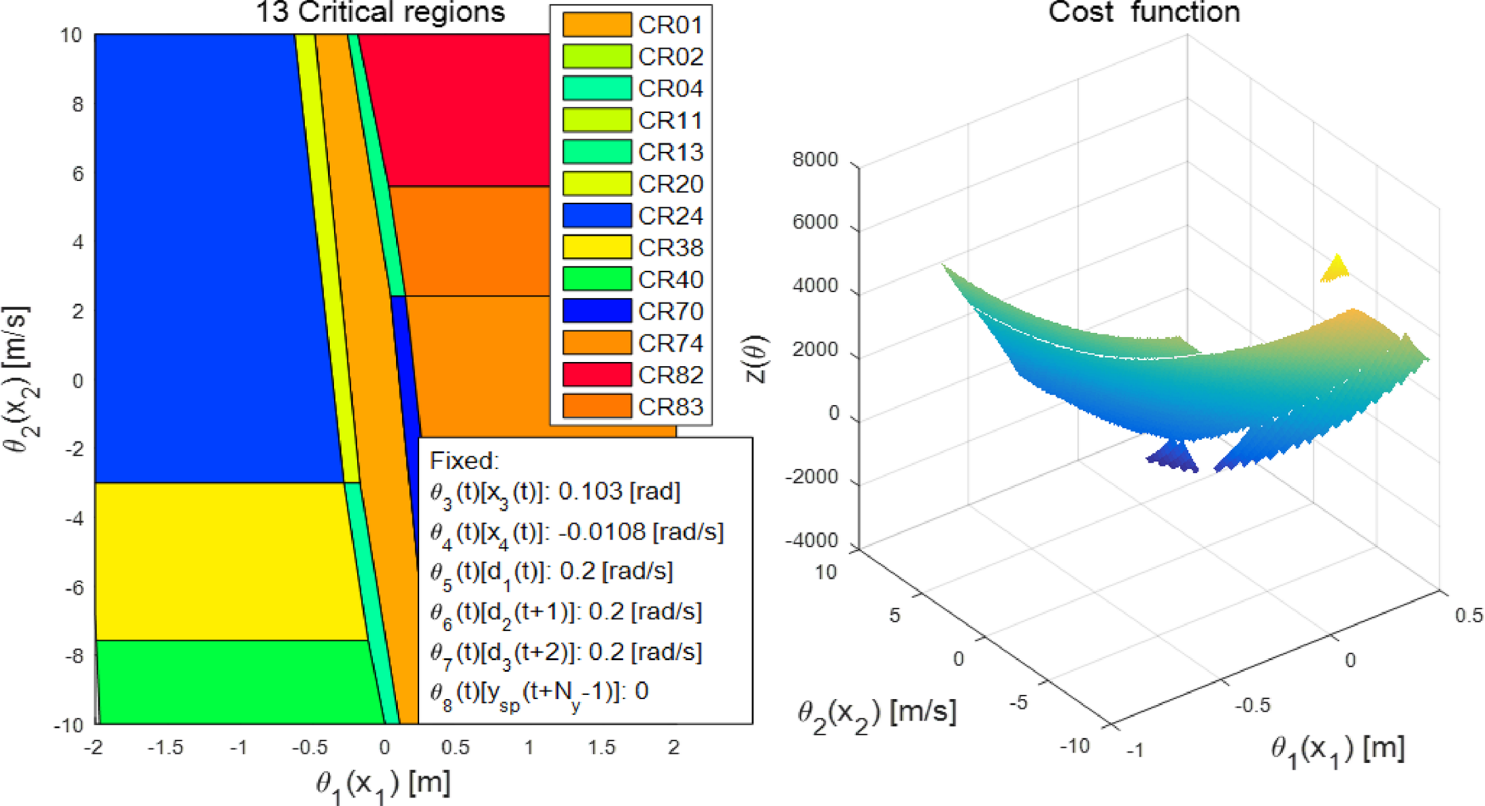
Critical regions. An example of critical regions and the associated cost function are illustrated in this figure. In this figure, $\theta \left(t\right)≜[x\left(t\right);\;\;u_{2}\left(t\right);\;\;u_{2}\left(t+1\right);\;\;u_{2}\left(t+2\right);\;\;y_{sp}\left(t+N_{y}-1\right)]$, where *x*(*t*), *u*_2_(*t*), and *y*_*sp*_(*t*) are the state vector, second input shown in Eq (2), and set point of the output within the prediction horizon *N*_*y*_, respectively. The proposed controller predicts the second input, which can be obtained from the desired path, along *N*_*y*_.

Fig 3 shows an example of the generated critical regions and the cost function of the parameter space using the POP solver, where $\theta \left(t\right)≜\left[\begin{array}{ccccc} x(t); & u_{2}\left(t\right); & u_{2}\left(t+1\right); & u_{2}\left(t+2\right); & y_{sp}\left(t+N_{y}-1\right) \end{array}\right]$. *u*_2_(*t*) and *y*_*sp*_(*t*+*N*_*y*_ − 1) indicate the predicted second inputs and the set point of the output at time *t* during the prediction along *N*_*y*_, respectively, where the value of *u*_2_(*t*) can be obtained from the geometric information of the desired path. In Fig 3, it is assumed that *N*_*y*_ = 3 and *N*_*u*_ = 2; correspondingly, a total of 97 critical regions are generated. In addition, *x*_1_(*t*) and *x*_2_(*t*) vary with the constraint on each state, whereas other values are fixed to specific values observed in the simulation. A unique sequence of MPC feedback laws is imposed in each critical region, and the controller chooses a critical region considering the value of *θ*(*t*), which minimizes the cost function. For example, if *x*_1_(*t*) and *x*_2_(*t*) are zero in Fig 3, the selected critical region is *CR*_001_ and the feedback law is *u*_1_(*t*) = −*Kθ*(*t*), where $K=[\begin{array}{cccccccc} -69.00 & -2.73 & -55.09 & 0.26 & 0.30 & 0.05 & -0.08 & 0.36 \end{array}]$. The number of MPC feedback laws are identical to the range of *N*_*u*_ in a MPC scheme, and only the first MPC feedback law is employed for the control action to the system. Therefore, explicit MPC does not require any online optimization owing to the critical regions, and this significantly reduces the computational complexity of MPC.

## Controller design

This section presents a formulation of the target path and thedesign of an explicit MPC controller; for comparison, an LQR and an MPC controller are designed as well.

### Desired path

In this subsection, we study the desired path, as shown in Fig 1 according to its four parts.

The first part of the desired path is a 200-m-long straight part, wherein we intend to show the ability of the proposed controller to fulfil constraints that will be defined in the next section (explicit MPC controller design) by setting the starting point such that is deviated from the desired path. The result of doing so is provied in the result section. The second and the third parts are curve roads with a constant radius of curvature. The radius of second part, as shwon in Fig 1, is 200 m, and that of the third is 100 m. In these two curved parts, the desired yaw rate is defined as follows [19]:

$$\begin{array}{c} {\dot{\psi }}_{des}=\frac{V_{x}}{R}=kV_{x}, \end{array}$$ (10)

where *k* is the curvature of the road. In Eq (10), *R*(*t*) is the inverse of *k*(*t*); therefore, in the second and third parts, the desired yaw rates are -0.1 rad/s and 0.2 rad/s, respectively.

The last part is a clothoid. A clothoid is mainly used to translate the type of road, e.g., from a circular load to a straight road. In the clothoid part, the curvature shown in Eq (10) is a linear function of the length from the initial position of the curve. The equation of a clothoid can be defined in terms of the Fresnel integral [26] as follows:

$$\begin{array}{ccc} \;\;\;\;\;\;\;\;\;\;\;\;\;\left[\begin{array}{c} x_{global}\left(t\right)\\ y_{global}\left(t\right) \end{array}\right]=a\left[\begin{array}{c} C(t)\\ S(t) \end{array}\right], & & \\ \text{where} & & \\ \;\;\;\;\;\;\;\;\;\;\;\;\;\;C\left(t\right)=\int_{0}^{t}\text{cos}\left(\frac{\pi u^{2}}{2}\right)du\;\text{and}\;S\left(t\right)=\int_{0}^{t}\text{sin}\left(\frac{\pi u^{2}}{2}\right)du. & \end{array}$$ (11)

In Eq (11), *x*_*global*_(*t*), *y*_*global*_(*t*), and *a* are the global X coordinate, the global Y coordinate in Fig 1, and a scaling factor, and the parameter *t* is positive. The curvature of the initial point and end point of the clothoid part are 0.01, which is the curvature of the third part, and zero, respectively. In a clothoid curve, the curvature is defined as a linear function of time as $k\left(t\right)=\frac{\pi }{a}t$, where *a* = 8372.3.

### Explicit MPC controller design

This subsection explains the design of the explicit MPC controller such as the controller structure; the determination of weighting matrices *Q*, *R*, *QR*; and the set of the constraints in Eq (3). Moreover, the simulation results for the controller with variations in the prediction horizon and control horizon in the MPC optimization problem are provided in this section.

**Fig 4:**
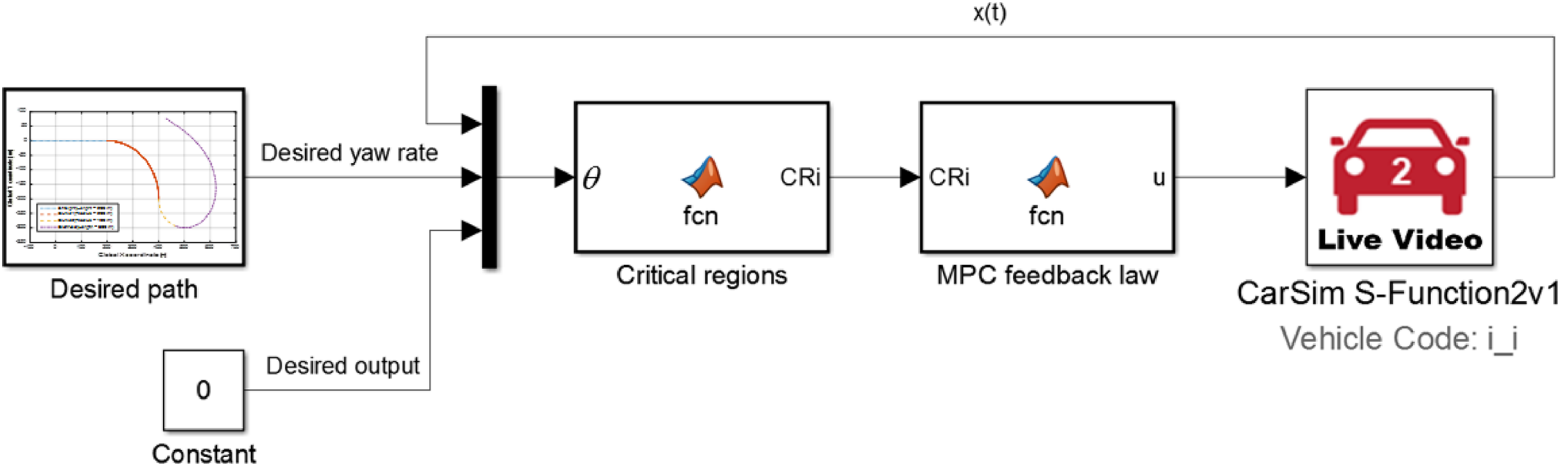
Explicit MPC controller structure. Structure of an explicit MPC controller for path-following control constructed using a vehicle model from CarSim. Based on the values of the parameter vector *θ*, the block “critical regions” selects a critical region *CRi*; then, the block “MPC feedback law” calculates the control action by applying the first MPC feedback law to the selected critical region *CRi*.

Fig 4 shows the structure of the explicit MPC controller using a vehicle model from CarSim(C-Class Hatchback 2017). The desired yaw rate ${\dot{\psi }}_{des}\left(t\right)$ can be obtained using Eq (10) along with the geometric information of the desired path. The desired output *y*_*des*_(*t*) and the set point of the output *y*_*sp*_(*t*) in the horizon *N*_*y*_ are fixed to zero because *y*(*t*) in Eq (2) represents the lateral deviation from the desired path. The value of the state vector *x*(*t*) is observed from the vehicle model in CarSim. Because this vehicle model reflects more realistic physical characteristics, which cannot be considered in Eq (2), which has only two degrees-of-freedom (DOF), a relatively more reliable demonstration of the proposed controller can be established.

For comparison of the proposed controller with other controllers, the constraints on the state vector, which consists of the error variables *e*_1_(*t*), *e*_2_(*t*) and the deviations of both *e*_1_(*t*) and *e*_2_(*t*) are defined as follows:

$$\begin{array}{c} \left[\begin{array}{c} -1\\ -10\\ -28.65\\ -572.96 \end{array}\right]\leq \left[\begin{array}{c} e_{1}\left(t\right)\;\left[\text{m}\right]\\ {\dot{e}}_{1}\left(t\right)\;\left[\text{m/s}\right]\\ e_{2}\left(t\right)\;\left[\text{deg}\right]\\ {\dot{e}}_{2}\left(t\right)\;\left[\text{deg/s}\right] \end{array}\right]\leq \left[\begin{array}{c} 1\\ 10\\ 28.65\\ 572.96 \end{array}\right]. \end{array}$$ (12)

This set of constraints considers the time when the starting position of the vehicle deviates from the desired path. In simulation results, the proposed controller shows the ability to fulfil these constraints to a greater extent compared with other optimization controllers.

**Fig 5:**
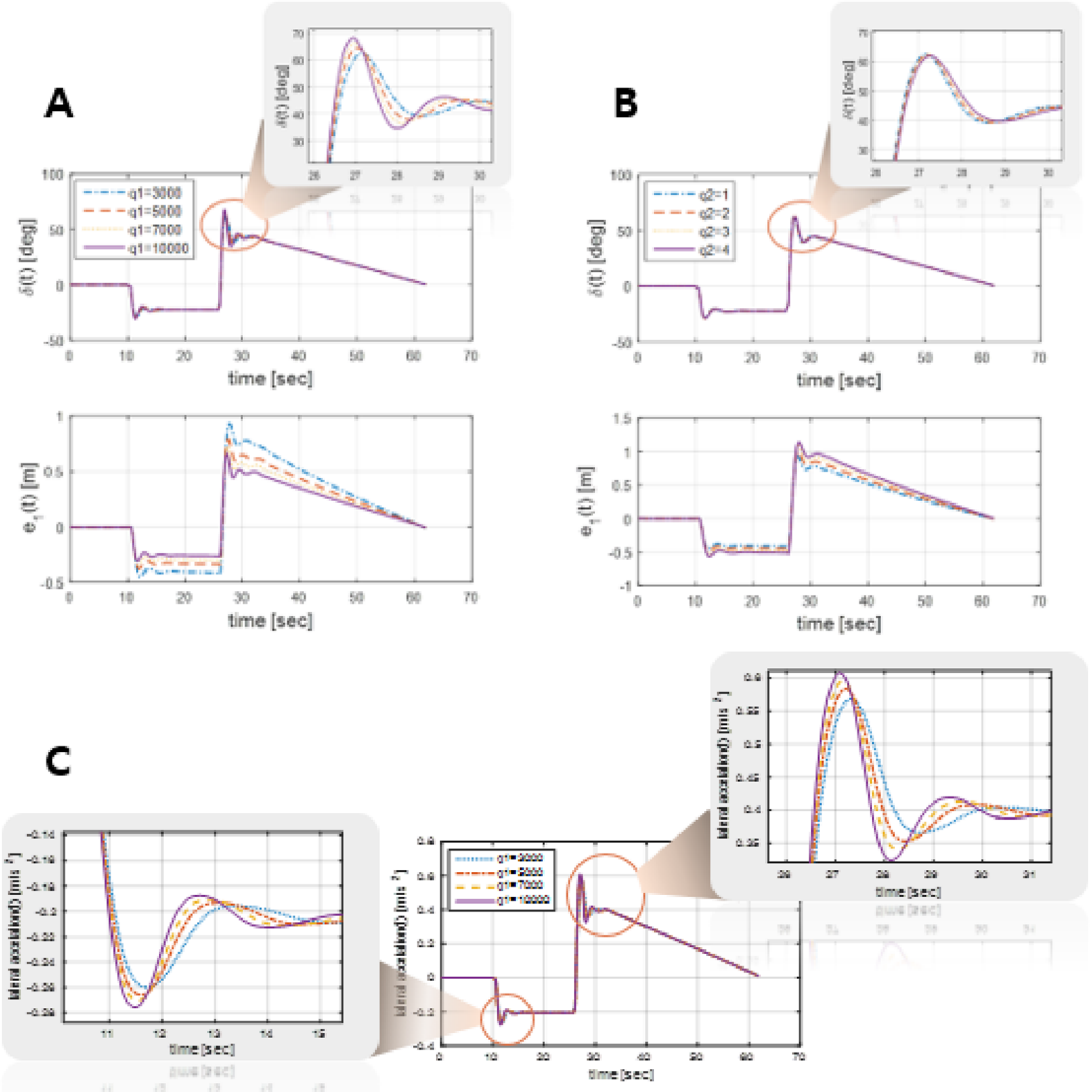
Determination of weighting factors for the state. (A) and (B) The effects of *q*_1_ and *q*_2_, respectively, which are weighting factors of the state, as given in Eq (13), where *e*_1_(*t*) indicates the lateral position error of the vehicle with respect to the desired path. It is demonstrated that for achieving path following control with error variables, the weighting factor of the position error must be large, whereas the weighting factor of the position error derivative must be small. (C) The lateral acceleration of the sprung mass with different values of *q*_1_. As ride comfort is typically evaluated according to the sprung mass of the vehicle, this figure shows a very large *q*_1_ deteriorates ride comfort.

The weighting matrices *Q*, *R*, and *QR* of the proposed controller are selected as follows:

$$\begin{array}{c} Q=[\begin{array}{cccc} q_{1} & 0 & 0 & 0\\ 0 & q_{2} & 0 & 0\\ 0 & 0 & q_{3} & 0\\ 0 & 0 & 0 & q_{4} \end{array}],R=bI,QR=cI, \end{array}$$ (13)

where *q*_1_, *q*_2_, *q*_3_, and *q*_4_, which are elements of the matrix *Q*, are the weighting factors for each state, and *b* and *c* are the weighting factors for the input and output errors, respectively. A relatively large weighting factor acts as a *hard* constraint on the state, whereas a relatively small weighting factor acts as a *soft* constraint on the state. Fig 5 shows the variations in each element in the matrix *Q*, where Fig 5A and 5B express the steering wheel angle *δ*(*t*) and the corresponding lateral position error *e*_1_(*t*), respectively, according to different values of *q*_1_ and *q*_2_. It can be proved that for position and angle error control, a relatively large weighting factor, which serves as a hard constraint, needs to be assigned to the position error variable, and a relatively small weighting factor, which serves as a soft constraint, is recommended for the derivative of the position error variable. However, it can be found in Fig 5C that a vary large weighting factor of the lateral position error leads to an increase in the maximum angle and a rapid changes in the steering wheel angle, which correspondingly increases the lateral acceleration of the sprung mass of the vehicle. It is generally accepted that a big sprung mass acceleration level causes deterioration of ride comfort [27]. In terms of the relationship between weighting matrices and performance, the weighting factors in Eq (13) are set to *q*_1_ = 7000, *q*_2_ = 1, *q*_3_ = 20000, *q*_4_ = 1, *b* = 1, and *c* = 100.

In [23], it is proved that for predictive controllers, the length of a prediction horizon must be longer than or equal to that of an input horizon. The lengths of the prediction horizon *N*_*y*_ and the input horizon *N*_*u*_ are analyzed in this section.

**Fig 6:**
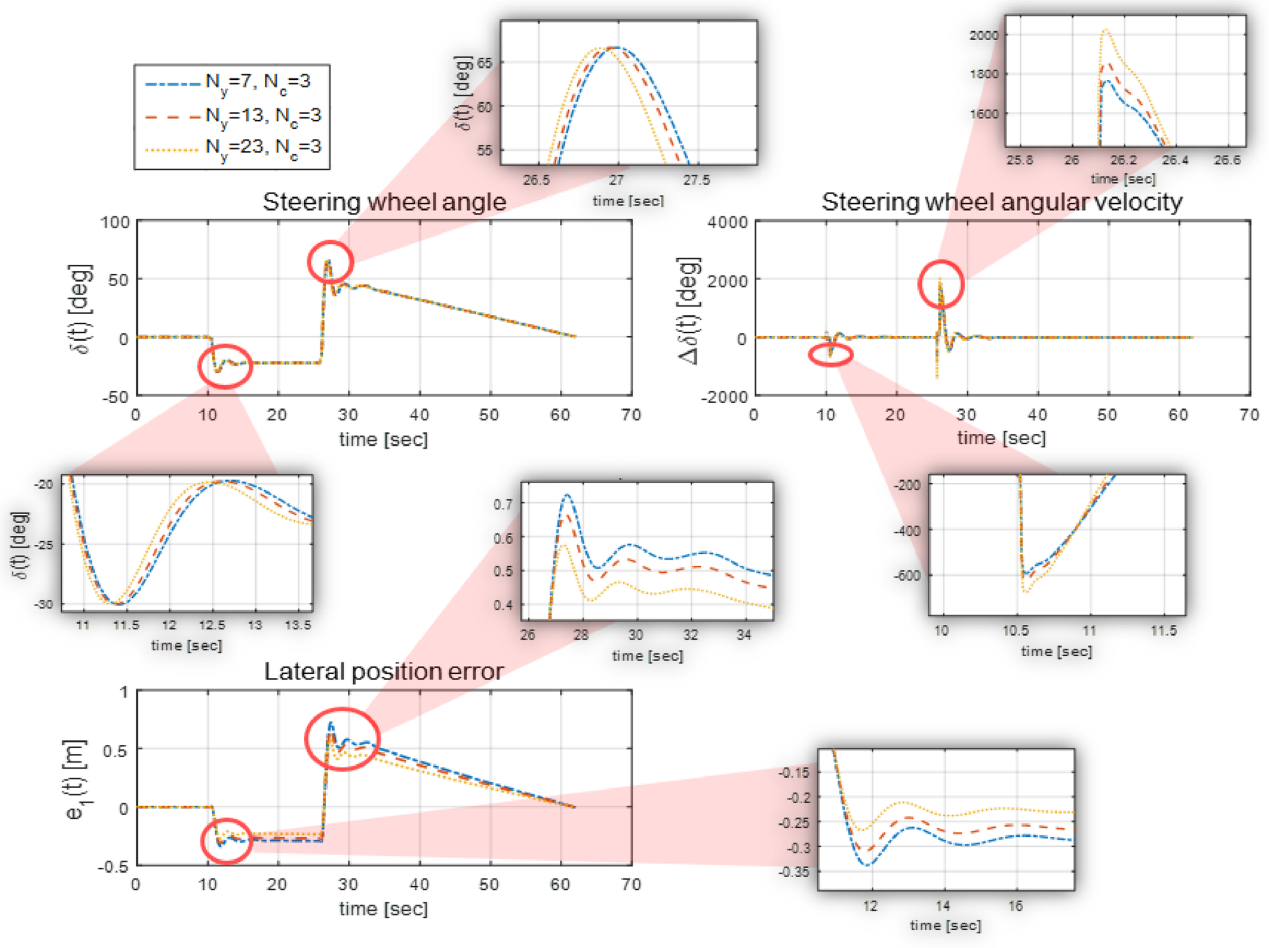
Simulation results with different ranges of prediction horizon. This figure shows the simulation results when the range of the prediction horizon *N*_*y*_ is varied while the input horizon *N*_*u*_ is fixed at 3. As *N*_*y*_ increases, the input dynamics, i.e., the steering wheel angle, changes in advance; this consequently reduces the lateral position error because a longer *N*_*y*_ improves the prediction ability of the controller. However, we found that an extremely long *N*_*y*_ leads to an increase in the steering wheel angular velocity, which deteriorates ride comfort.

Fig 6 shows the optimal steering wheel angle and the generated lateral position error of the proposed controller, when *N*_*y*_ is varied. In Fig 6, *N*_*c*_ is fixed to 3 and *N*_*y*_ is varied to 7, 13, and 23. As the simulation results show, the change in the steering wheel angle occurs earlier as *N*_*y*_ increases, which reduces the lateral position error. Similarly, the ability of the explicit MPC controller to anticipate future events can be improved if the length of *N*_*y*_ is increased. However, it is found that the steering wheel angular velocity increases as *N*_*y*_ increases. This means that setting a vary long prediction horizon to reduce the lateral error will deteriorate ride comfort. Therefore, considering the two results from Fig 6, the prediction horizon and the input horizon were set to *N*_*y*_ = 11 and *N*_*c*_ = 3.

## Results

In this section, the performance of the proposed controller is compared with those of the LQR controller and the MPC controller. The actual driving simulation results obtained using CarSim are addressed as well.

In the design of the LQR controller and the MPC controller, the weighting matrices used are the same as those used in case of the proposed explicit MPC controller. The state feedback gain *K* of the LQR controller can be obtained using the algebraic Riccati equation as follows [28]: Therefore, *K* is the same as shown in Eq (4). The MPC controller solves the optimization problem Eq (3) online considering the constraints given in Eq (12).

**Fig 7:**
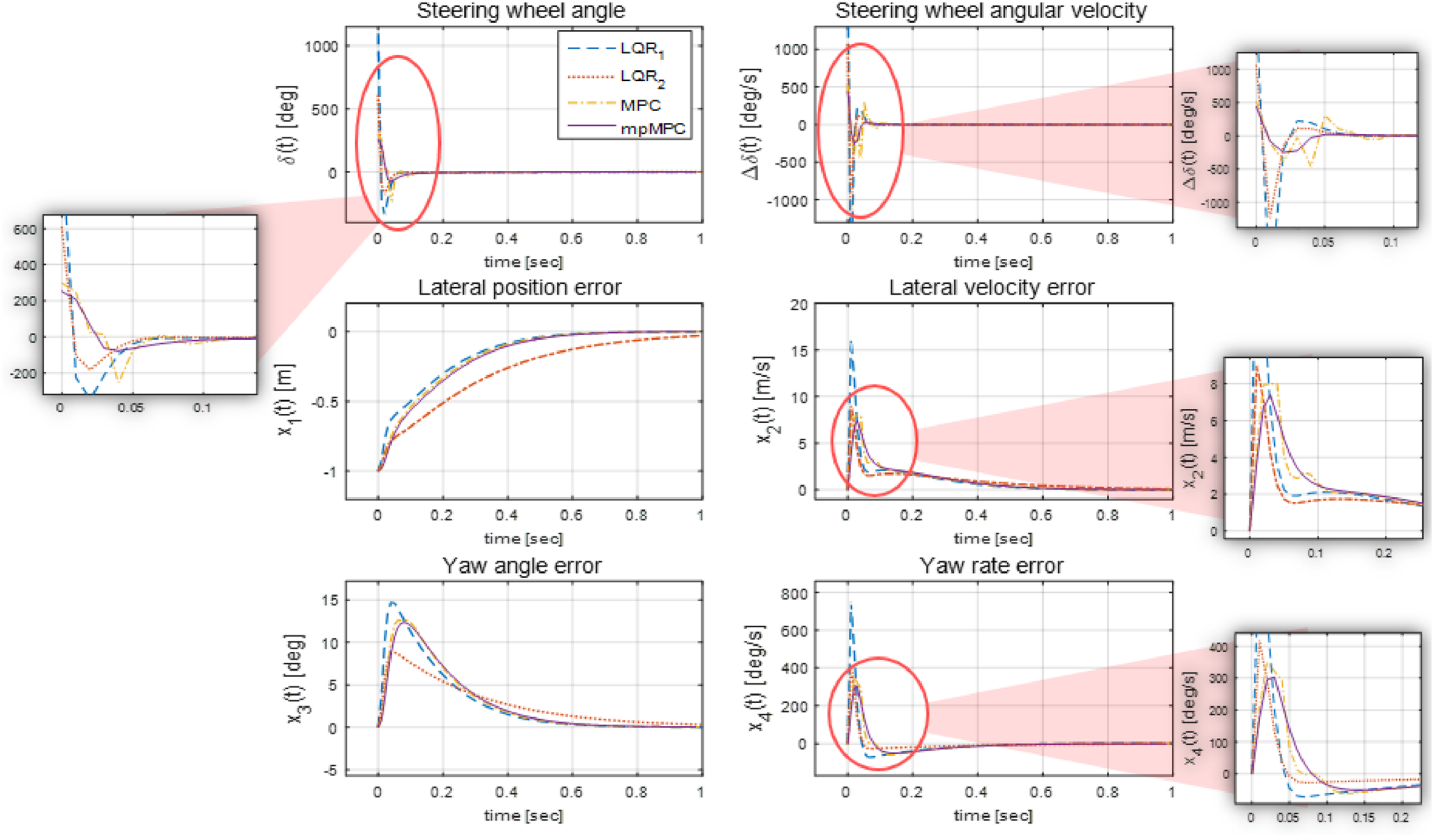
Comparison of optimization controllers. This figure shows the dynamics of the states of the LQR controllers, MPC controller, and explicit MPC controller. It is proved that LQR_1_ cannot fulfil the constraints as set Eq (12) and that the MPC controller consumes more time than the explicit MPC controller in the first 100 simulation runs (0.51 s in the case of the explicit MPC controller and 35.71 s in the case of the MPC controller). Moreover, LQR_2_ is designed to limit the maximum values of the state dynamics in the constraints by adjusting the weighting matrices; nevertheless, a high steering wheel angular velocity, which reduces ride comfort, persist.

Fig 7 shows the simulation results of two different LQR controllers, the MPC controller, and the explicit MPC controller. It is assumed that the vehicle starts with 1 m of lateral position error, and these controllers calculate the optimal steering angle to lead the vehicle along the desired path. In the case of LQR_1_, the weighting matrices *Q*, *R*, *P*, and *QR* are the same as those in the explicit MPC controller design, and they cannot fulfil the constraints given in Eq (12). Therefore, LQR_2_ was additionally designed to confine the dynamics of the states to the constraints by adjusting the weighting matrices, specifically, to reduce *q*_1_ in the weighting matrix *Q*. Even though the states change within the constraints, the steering wheel angular velocity at t = 0 remains too high, which degrades ride comfort. Moreover, adjustment of the matrices deteriorates the tracking ability to converge the error variables to zero. However, the MPC controller, designed using YALMIP toolbox, and the explicit MPC controller, designed using the POP solver, induce the optimal input while fulfilling the constraints. However, the main difference between the MPC controller and the explicit MPC controller is in terms of the time required to solve the optimization problem. In the first second of the simulation, the MPC controller required 35.71 s to solve optimization problems online, whereas the explicit MPC controller required only 0.51 s owing to its use of critical regions to explicitly choose the optimal feedback law.

**Fig 8:**
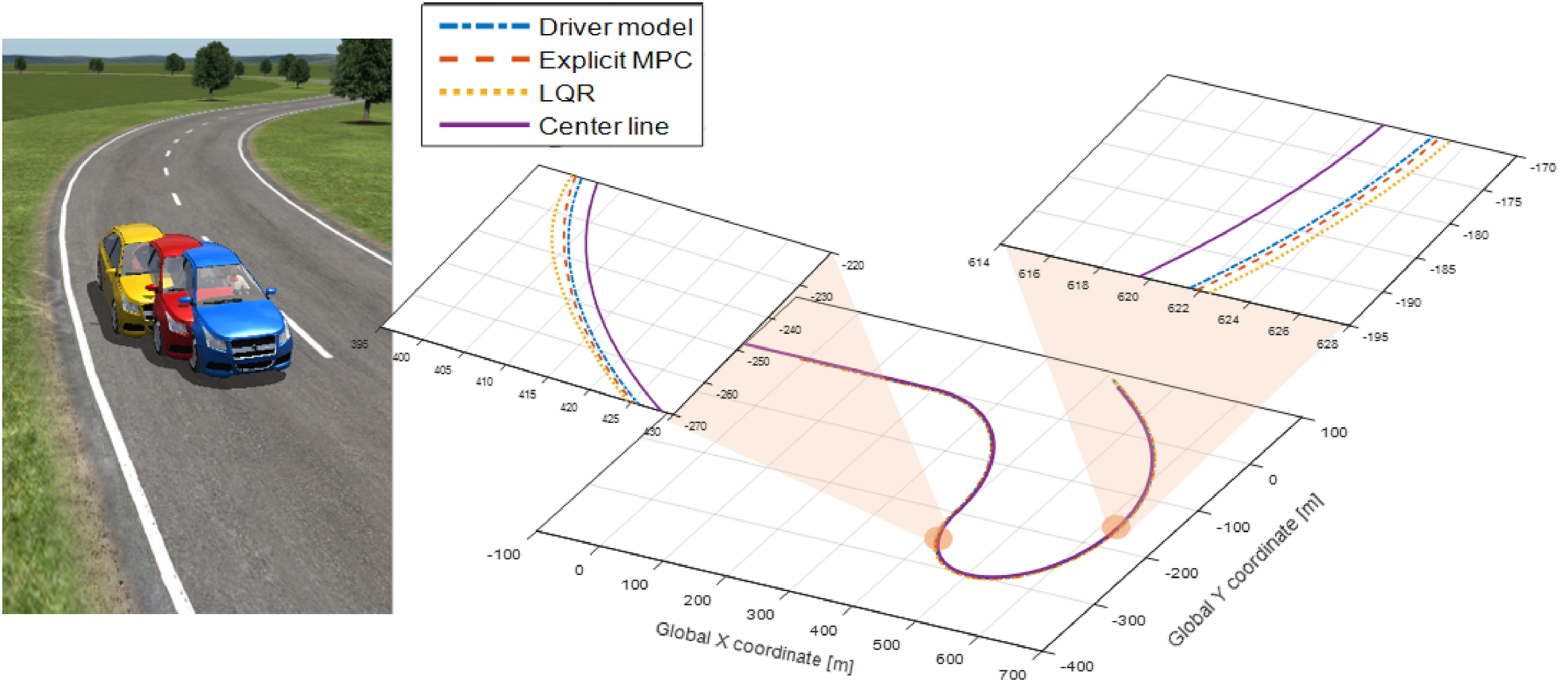
Paths of controllers and driver model. This figure shows the paths of the LQR controller, explicit MPC controller, and driver model. It can be observed that in the case of the LQR controller, the deviation from the center line of the desired path is larger than that in the case of the explicit MPC controller, whereas path-following control performed using the explicit MPC controller is similar to that performed using the driver model in CarSim. The details of the error variables are shown in Fig 9.

Fig 8 shows the paths of the LQR controllers, explicit MPC controller, and driver model from CarSim. The objective of path-following in this simulation is to maintain 2 m of lateral distance from the center line of the desired path. As shown in the figure, the path of the LQR controller causes a positional deviation from the desired path, and this deviation is larger than that in case of the explicit MPC controller. Conversely, the explicit MPC controller is capable of path-following because its path is close to the that of the driver model.

**Fig 9:**
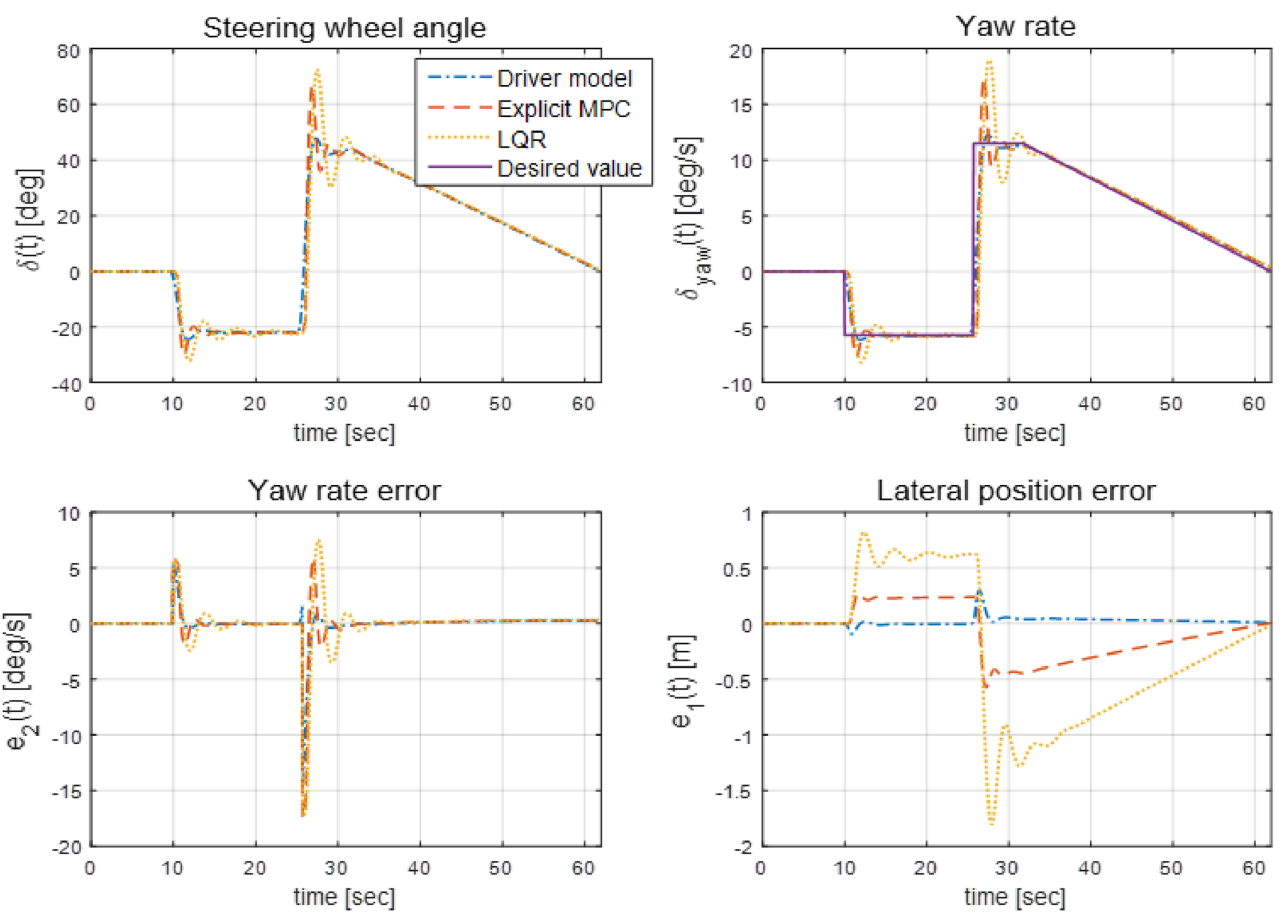
Simulation results for optimization controllers and the driver model. Simulation results for the path-following controllers obtained using the LQR and explicit MPC methods as well as those obtained using the driver model are shown in this figure. Both controllers use the same weighting matrices to solve the optimization problem. From the results of the error variables, in particular, from the result of the lateral position error, the superiority of the explicit MPC controller over the LQR controller is demonstrated.

The dynamics of the steering wheel angle, yaw rate, and lateral position error are shown in Fig 9. The dynamics of the three states are bounded in the permitted ranges, which are set using the constraints Eq (12), whereas the range of the lateral position error of the LQR controller is out of the boundary set. Based on these simulation results, the state-space representation expressed in Eq (2) and the tire cornering stiffness value determined from Fig 2 can be verified because CarSim can handle such complex dynamics of vehicles, which cannot be considered in Eq (2).

**Fig 10:**
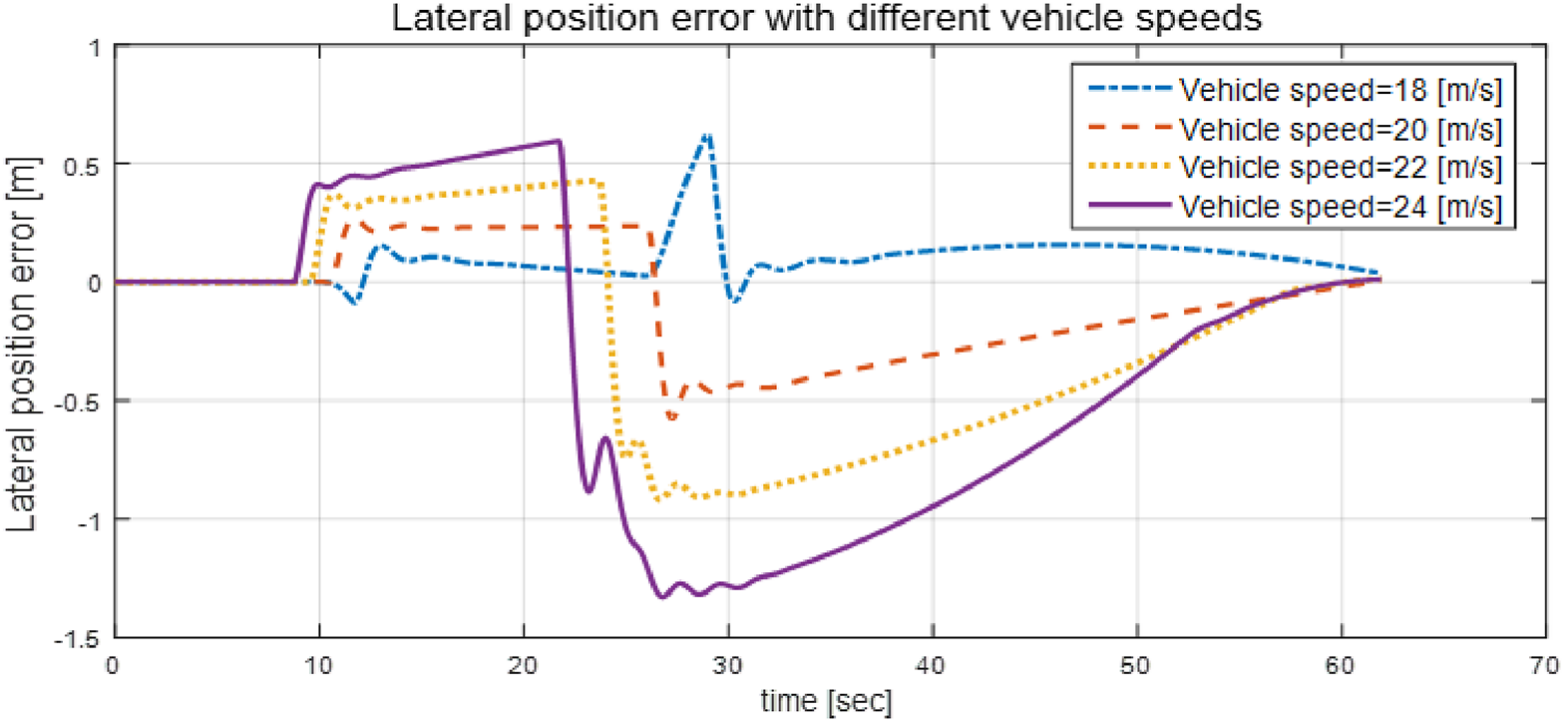
Lateral position errors at different vehicle speeds. This figure shows the lateral position error that occurred when the vehicle speed was 18 m/s, 20 m/s, 22 m/s, and 24 m/s. The position error increases as the vehicle speed increases.

Additionally, simulations, were performed by varying the vehicle speed to 18 m/s, 20 m/s, 22 m/s, and 24 m/s, and the results are shown in Fig 10. As can be explained from Fig 10, the positional deviation from the desired path increases as the vehicle speed increases. This problem can be handled by increasing the weighing factor of the state of the lateral position error, as proved in Fig 5A, as long as the corresponding increase in steering wheel angle dose not severely degrade ride comfort.

## Conclusion

In this study, an explicit MPC controller for path-following control was designed and analyzed using MATALB/Simulink and CarSim. Explicit MPC has been proposed to reduce the computational complexity caused by the online optimization of MPC. Explicit MPC generates critical regions, by using a multi-parametric quadratic programming technique, so that the controller can explicitly obtain the optimal feedback gain. The explicit MPC scheme, which is a method of determining the weighting matrices, and the range of the prediction horizon and the input horizon for path-following control were described in this paper. The tracking ability and fulfilment of the constraints of explicit MPC were proved comparing its performance with those of other controllers.

In the future works, we aim to design an explicit MPC controller for path-following control where a vehicle model considers road profile excitation. For example, a vehicle model can be combined with a three-dimensional (3-D) road profile excitation, which has been presented in [29]. One of the main contributions of this paper is to prove the performance of explicit MPC controllers, which can reduce the computational complexity of MPC so that the MPC scheme can be applied for relatively faster and/or smaller problems. For this purpose, we intend to design an explicit MPC controller on Field Programmable Gate Array(FPGA) using VHSIC Hardware Description Language(VHDL) for the future work. FPGA is based around a matrix of configurable logic blocks, which are connected via programmable interconnects. VHDL is a hardware language used for simulation of electronic designs such as FPGA. Because the MPC-based controllers needs a relatively huge computational effort, most of MPC controllers are designed on MCUs. Compared with MCUs, however, FPGA features the lower development coasts and lower power consumption; especially, by using high-speed CMOS technology, this device can handle relatively faster or smaller problems than MCUs generally do [30]. However, it has been known that FPGA is unable to complete a complex design such as the online optimization problem in the MPC scheme. Regarding this issue, the explicit MPC scheme only needs simple mathematical calculations to choose a critical region, which means FPGA can be used to design explicit MPC controllers. We ultimately aim to design explicit MPC controllers for not only path-following control but also UAVs or other automotive systems such as electric vehicle powertrain and braking control systems [31–34]. The important characteristics of controllers for both UAVs and automotive systems are the low power consumption and the fast response to the control action. We intend to prove that explicit MPC controllers can be applied for both of the target applications while satisfying these two characteristics by using FPGA.

## Supporting information

S1 Dataset Constraints for partitioning of critical regions when the prediction horizon is 3 and input horizon is 2. (XLSX)

S1 Table Detailed information about explicit controllers with different prediction and input horizons. (DOCX)
