# Supplementary material for: Analysis of explicit model predictive control for path-following control
Source: PLoS One. 2018 Mar 13;13(3):e0194110. doi: 10.1371/journal.pone.0194110 (PMC5849315; doi:10.1371/journal.pone.0194110)
Supplement: S1 Table — (DOCX) [file pone.0194110.s002.docx]

**S1 Table.** Detail information of explicit controllers with different prediction and input horizons

| Prediction horizon | Input horizon | Number of critical regions | Elapsed time for 100 simulations [sec] | |
| --- | --- | --- | --- | --- |
|  |  |  | Explicit MPC | MPC |
| 3 | 1 | 15 | 0.03 | 32.52 |
| 3 | 2 | 63 | 0.08 | 33.74 |
| 5 | 1 | 23 | 0.04 | 33.48 |
| 5 | 2 | 129 | 0.10 | 33.23 |
| 5 | 3 | 245 | 0.18 | 33.77 |
| 5 | 4 | 415 | 0.3 | 33.81 |
| 7 | 2 | 189 | 0.16 | 34.04 |
| 7 | 3 | 421 | 0.29 | 35.16 |
| 7 | 4 | 795 | 0.49 | 34.59 |
| 7 | 5 | 1347 | 0.77 | 35.80 |

Explicit MPC allows the controller to solve an optimization problem offline by using a multi-parametric quadratic programming technique. As this table shows, the total elapsed time for the simulations of the explicit MPC controller is a lot shorter than of the MPC controller. In addition, the number of critical regions of the explicit MPC controller is increased if either the prediction horizon or the input horizon increases.
